# In Situ Targeting RGD-Modified Cyclodextrin Inclusion Complex/Hydrogel Hybrid System for Enhanced Glioblastoma Therapy

**DOI:** 10.3390/pharmaceutics17070938

**Published:** 2025-07-20

**Authors:** Xiaofeng Yuan, Zhenhua Wang, Pengcheng Qiu, Zhenhua Tong, Bingwen Wang, Yingjian Sun, Xue Sun, Lu Sui, Haiqiang Jia, Jiajun Wang, Haifeng Tang, Weiliang Ye

**Affiliations:** 1 Department of Pharmaceutics, School of Pharmacy, Fourth Military Medical University, Xi’an 710032, China; xiaofeng012345@163.com (X.Y.); sunyingjian2024@163.com (Y.S.); 13233030861@163.com (H.J.); 15319782276@163.com (J.W.); 2 Department of Chinese Materia Medical and Natural Medicines, School of Pharmacy, Fourth Military Medical University, Xi’an 710032, China; qpc023@fmmu.edu.cn (P.Q.); wbw134610@163.com (B.W.); 3 Chinese People’s Liberation Army Logistics Support Force No. 967 Hospital, Dalian 116021, China; 4 General Hospital of Northern Theater Command, No. 83, Wenhua Road, Shenyang 110016, Chinasunxue88@163.com (X.S.);

**Keywords:** doxorubicin, cyclodextrin, inclusion complex, hydrogel, glioblastoma

## Abstract

**Background/Objectives**: Glioblastoma (GBM) remains the most aggressive primary brain tumor, characterized by high malignancy, recurrence rate, and dismal prognosis, thereby demanding innovative therapeutic strategies. In this study, we report a novel in situ targeting inclusion complex hydrogel hybrid system (DOX/RGD-CD@Gel) that integrates doxorubicin (DOX) with RGD-conjugated cyclodextrin (RGD-CD) and a thermosensitive hydrogel for enhanced GBM therapy. **Methods**: The DOX/RGD-CD@Gel system was prepared by conjugating doxorubicin (DOX) with RGD-modified cyclodextrin (RGD-CD) and embedding it into a thermosensitive hydrogel. The drug delivery and antitumor efficacy of this system were evaluated in vitro and in vivo. **Results**: In vitro and in vivo evaluations demonstrated that DOX/RGD-CD@Gel significantly enhanced cytotoxicity compared to free DOX or DOX/CD formulations. The targeted delivery system effectively promoted apoptosis and inhibited cell proliferation and metastasis in GBM cells. Moreover, the hydrogel-based system exhibited prolonged drug retention in the brain, as evidenced by its temperature- and pH-responsive release characteristics. In a GBM mouse model, DOX/RGD-CD@Gel significantly suppressed tumor growth and improved survival rates. **Conclusions**: This study presents a paradigm of integrating a targeted inclusion complex with a thermosensitive hydrogel, offering a safe and efficacious strategy for localized GBM therapy with potential translational value.

## 1. Introduction

Glioblastoma (GBM), one of the most malignant primary brain tumors, presents a significant threat to patients’ lives and health due to its high incidence, recurrence rates, and notably poor prognosis [1,2]. The median survival duration of GBM patients is alarmingly short at 12–15 months, with a 5-year survival rate <5%, and the quality of life is significantly impaired [3]. Over the years, comprehensive treatment strategies for GBM, including surgical intervention, radiotherapy, and chemotherapy, have been continuously refined. However, the outcomes of these treatments have fallen short of expectations, leaving much to be desired [4,5]. Consequently, there is an urgent need and a pressing research priority to develop novel, efficient, targeted GBM treatment methods with low toxicity.

In this regard, chemotherapeutic agent doxorubicin (DOX) emerges as a promising candidate for GBM treatment due to its broad-spectrum antitumor activity. Although DOX has been explored for GBM therapy in preclinical studies, its clinical application remains limited due to significant systemic toxicity and off-target effects [6,7]. As DOX is classified as a Biopharmaceutics Classification System (BCS) class III drug, its bioavailability is primarily limited by permeability rather than dissolution. To address these limitations, researchers have been actively exploring strategies to encapsulate DOX within carriers, aiming to achieve high local concentrations of the drug in tumor tissues through targeted delivery. This strategy holds the potential to enhance therapeutic efficacy while reducing systemic toxicity [8,9,10], thereby offering a more viable and effective treatment approach for GBM patients.

Cyclodextrin (CD) and its derivatives have attracted considerable attention as drug carriers, primarily attributed to their unique cavity structure and excellent biocompatibility [11,12,13]. The cavities of CD can effectively encapsulate drug molecules, forming stable inclusion complexes. This encapsulation process not only shields the drugs from external factors that may cause degradation but also improves their stability and bioavailability. Furthermore, by chemically modifying CD with specific targeting molecules, such as the Arg–Gly–Asp (RGD) peptide (Figure S1), it becomes possible to enhance the targeting ability of the drugs towards specific tumor cells [14,15]. Such a targeted delivery strategy can effectively reduce drug distribution in normal tissues, thereby significantly lowering systemic toxicity and improving treatment outcomes.

The short RGD peptide exhibits remarkable specificity in binding to various integrins, making it a valuable tool in the design of tumor-targeted drugs due to its exceptional targeting effects and biological activity [16,17]. Integrins, which serve as critical receptors on the cell membrane, play a pivotal role in vital cellular processes such as adhesion, migration, and signal transduction. Notably, such integrins as αvβ3 and αvβ5 are overexpressed in numerous tumor cells [18,19]. By modifying drug carriers with the RGD peptide, drugs can achieve specific recognition and binding to tumor cells, thereby enhancing the targeted delivery and bioavailability of the drugs while minimizing their damage to normal tissues.

In recent years, thermosensitive hydrogels have attracted significant attention as an innovative class of drug delivery carriers, primarily due to their superior biocompatibility and injectability [20,21,22]. Upon injection, thermosensitive hydrogels rapidly solidify into a semisolid state at body temperature. This rapid solidification enables the hydrogels to stabilize quickly after administration, effectively preventing the rapid diffusion and clearance of drugs from the target site. Furthermore, the network structure of these hydrogels allows for the loading of a large number of drug molecules and facilitates sustained drug release. This sustained release mechanism prolongs drug retention in tumor tissues, increases local drug concentration, and ultimately enhances therapeutic efficacy [23,24]. Therefore, by integrating hydrogels with inclusion complex-based drugs, it becomes feasible to construct a drug delivery system that combines sustained release and targeting properties. This innovative approach presents a promising strategy for the local treatment of GBM.

**Scheme 1:**
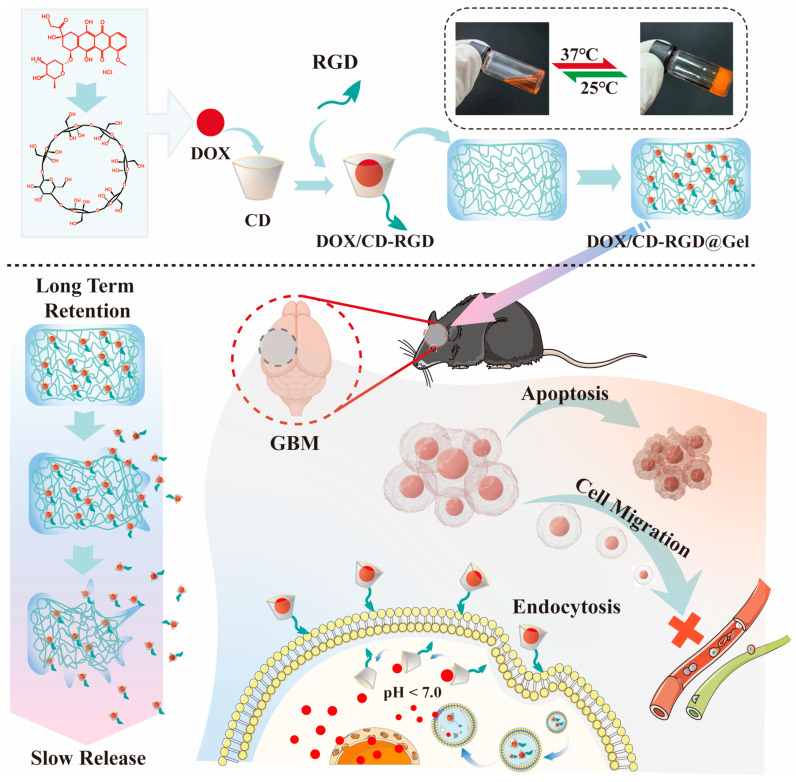
Formation and combined antiglioma mechanism of DOX/RGD-CD@Gel.

With these considerations in mind, this study seeks to develop a thermosensitive hydrogel, DOX/RGD-CD@Gel, modified with RGD for the local targeted treatment of GBM (Scheme 1). Leveraging the targeting effect of RGD, this hydrogel can specifically recognize and bind to GBM cells. Meanwhile, by utilizing the sustained drug release characteristics of thermosensitive hydrogels, the hydrogel can prolong drug retention in tumor tissues. These features collectively contribute to improving the bioavailability and therapeutic effects of DOX. This study not only provides a new, efficient, low-toxicity, local targeted treatment method for GBM, but also offers novel insights and approaches that may be applicable to the treatment of other malignant tumors.

## 2. Materials and Methods

### 2.1. Materials, Cell Lines, and Mice

Doxorubicin hydrochloride was purchased from Hisun Pharmaceutical Co., Ltd. (Taizhou, China), while carboxymethyl-β-cyclodextrin was obtained from MACKLIN Co., Ltd (Shanghai, China), and the RGD peptide was purchased from GLBiochem Co., Ltd. (Shanghai, China). N-hydroxysuccinimide (NHS) and 1-ethyl-3-(3-dimethylaminopropyl)carbodiimide (EDCI) were procured from Medpep Co., Ltd. (Shanghai, China), and poly(lactic-co-glycolic acid) (PLGA)–polyethylene glycol (PEG)–PLGA (Mn: PLGA blocks, 1500–2000 Da, PEG block, 1000–1500 Da; GA:LA = 50:50) was purchased from Tanshui Technology Co., Ltd. (GuangZhou, China). The hydrogel undergoes sol–gel transition at 37 °C. All other chemical reagents were of analytical grade and were obtained from commercial reagent suppliers. The anti-E-cadherin, anti-N-cadherin, anti-cleaved caspase-3, anti-Bax, and anti-Bcl-2 antibodies were purchased from Abcam Co., Ltd. (Cambridge, UK). Additionally, GL261 and HUVEC cells were obtained from Green Biotechnology Co., Ltd. (Xi’an, China). Male BALB/c mice were provided by the Animal Center of the Fourth Military Medical University. All animal experiments were conducted in strict accordance with the guidelines approved by the Ethics Committee of the Fourth Military Medical University (Xi’an, China).

### 2.2. Preparation and Characterization of DOX/RGD-CD

First, 306.8 mg of CM-β-CD was dissolved in 8 mL of deionized water in a 50 mL round-bottom flask, after which 46.1 mg of EDCI and 34.5 mg of NHS were added, and the mixture was stirred for 1 h. Afterwards, 150 mg of RGD was added, and the mixture was stirred for 24 h, dialyzed overnight, and then freeze-dried to obtain RGD-CD. Furthermore, the inclusion complex was prepared via the saturated aqueous solution method [25]. We dissolved 80 mg of RGD-CD in 500 μL of deionized water and dissolved 30 mg of free doxorubicin in 700 μL of anhydrous ethanol; then, we slowly mixed and stirred these solutions for three hours, and then centrifuged and washed the mixture with ethanol to obtain DOX/RGD-CD. DOX/CD can be prepared in the same way. The FTIR spectra were recorded using a Nicolet iS50 spectrometer (Thermo Fisher Scientific) with the KBr pellet technique. The number of scans was set to 32, and the spectral range was 400–4000 cm^−1^. NMR spectra were measured using a Bruker AVANCE III 400 MHz spectrometer, using deuterated dimethyl sulfoxide (DMSO-d_6_) as the solvent for ^1^H NMR, with 64 scans and a relaxation delay of 2 s.

### 2.3. In Vitro Cytotoxicity Study

The cytotoxicity of DOX/RGD-CD was evaluated via the MTT method. GL261 cells were seeded into a 96-well plate at a density of 4 × 10^3^ cells/well. After 24 h, the medium was replaced with different concentrations of drugs. After incubation for 48 h, an MTT solution was added to the wells for 4 h of incubation, after which 200 μL of dimethyl sulfoxide (DMSO) was added to each well. The absorbance at 490 nm was then measured using a microplate reader (Thermo Multiskan MK3, Waltham, MA, USA).

### 2.4. Live/Dead Cell Assay

The live/dead cell assay was performed according to the instructions of the Thermo Scientific kit. GL261 cells were cultured overnight in a 6-well plate (3 × 10^5^ cells/well) and then treated with different drug solutions. After incubation, fluorescence microscopy was used to photograph the cells and perform analysis.

### 2.5. Apoptosis Assay

Flow cytometry was utilized to evaluate drugs that induce apoptosis in tumor cells. GL261 cells were seeded into a 6-well plate (500 cells/well) and cultured for 24 h. The cell culture medium was then replaced with a fresh serum-free medium containing different drug solutions. After a 24 h co-culture with different treatments, the culture supernatant and cells were collected. The samples were centrifuged and incubated with 1× annexin binding buffer, and then detected by flow cytometry.

### 2.6. Expression of Apoptosis-Related Proteins in GL261 Cells

GL261 cells were inoculated into 6-well plates (1 × 10^6^ cells/well) and cultured for 24 h. The cell culture medium was replaced with a fresh serum-free medium containing different drug solutions, and the cells were incubated for 24 h. The cells were then collected, and the proteins were extracted. Western blotting was used to detect the expression of Bcl-2, cleaved caspase-3, and Bax in GL261 cells.

### 2.7. Cell Migration and Invasion Assay

For the cell migration assay, GL261 cells were inoculated into the transwell donor chamber (8 × 10^4^ cells/well) and cultured for 4 h. For the cell invasion assay, the donor chamber of the transwell was precoated with Matrigel, and GL261 cells were added (8 × 10^4^ cells/well). After the GL261 cells were cultured for 4 h, the medium in the donor chamber was replaced with a fresh serum-free medium containing different concentrations of drugs, and the cells were incubated for 24 h. The donor and recipient chambers were separated, and the cells inside the donor chamber were wiped with a cotton swab. Furthermore, the cells outside the donor chamber were fixed with 4% paraformaldehyde and stained with 0.1% crystal violet solution. Then, these cells were observed under a fluorescence microscope. One milliliter of 33% acetic acid solution was added to the donor chamber, and the absorbance of the acetic acid solution in the donor chamber was measured at 570 nm with a molecular device. Finally, the migration and invasion rates were calculated separately.

### 2.8. Expression of Invasion-Related Proteins in GL261 Cells

GL261 cells were inoculated into 6-well plates (1 × 10^6^ cells/well) and cultured for 24 h. The cell culture medium was replaced with a fresh serum-free medium containing different drug solutions, and the cells were incubated for 24 h. The cells were then collected, the proteins were extracted, and Western blotting was used to detect the expression of E-cadherin and N-cadherin.

### 2.9. Evaluation of In Vitro Uptake

GL261 cells were added to 24-well plates. After incubation for 24 h, the cell culture medium was replaced with a fresh serum-free medium containing different drug solutions, the cells were incubated for 30 min or 2 h, and the medium was discarded. The cells were subsequently washed three times with phosphate-buffered saline (PBS) at 37 °C and fixed with 4% paraformaldehyde for 15 min. DAPI solution (500 μL, 500 nmol/L) was added to stain the cell nuclei, after which the cells were washed three times with PBS and mounted on slides. Drug uptake by GL261 cells was observed via confocal laser scanning microscopy (CLSM; FV3000, Olympus, Tokyo, Japan). The cellular uptake of DOX/RGD-CD was also determined using a Waters 2695/2996 high-performance liquid chromatography system (HPLC, Waters, Milford, MA, USA). In addition, to clarify the mechanism by which GL261 cells take up DOX/RGD-CD, different inhibitors were added during the experiments.

### 2.10. Preparation and Characterization of DOX/RGD-CD@Gel

PLGA–PEG–PLGA was added to PBS to a concentration of 150 mg/mL and incubated at 4 °C until it dissolved. Next, the solution was mixed with an equal volume of the DOX/RGD-CD solution with the final concentration of DOX of 10 mg/mL. The mixture was incubated at room temperature for 12 h to allow hydrogel formation. The hydrogel was subsequently characterized via cryo-scanning electron microscopy (SYST TA PRO 1156; Hitachi, Tokyo, Japan). The elastic and viscous moduli were measured via rheological tests (Bohlin Gemini; Malvern Panalytical, Malvern, Worcestershire, UK).

### 2.11. In Vitro Drug Release Assay

The in vitro release of DOX from DOX@Sol, DOX/CD@Gel, and DOX/RGD-CD@Gel was determined via dialysis. Two milliliters of the DOX/CD@Gel and DOX/RGD-CD@Gel solutions were added to dialysis bags, which were placed in 40 mL of PBS containing 1% (*v*/*v*) Tween 80 at pH 7.4 or 5.8 and stirred at 100 rpm and 37 °C. At each predetermined timepoint, 2 mL of the release medium was removed, and the same volume of fresh PBS was added back to the system. After the samples were collected, the extent of DOX release was analyzed via HPLC.

### 2.12. Establishment of the Glioblastoma Tumor Model

The in situ GBM mouse model was established according to a previously reported method [26]. Using a non-stereotactic device, luciferase-labelled GL261 cells (GL261-Luc cells; 1 × 10^8^ cells/mL, 5 μL) were injected into the right striata of the mice.

### 2.13. In Vivo Retention of DOX/RGD-CD@Gel

To study the in vivo retention of DOX/RGD-CD@Gel, DOX@Sol, DOX/CD@Gel, or DOX/RGD-CD@Gel was injected into glioma tumors. Then, an IVIS Spectrum Imaging System (PerkinElmer, Waltham, MA, USA) was used for fluorescence imaging on days 1, 5, and 10 after administration.

### 2.14. Tumor Treatment with DOX/RGD-CD@Gel In Vivo

The in situ GBM model mice were divided into four groups: saline, DOX@Sol, DOX/CD@Gel, and DOX/RGD-CD@Gel. The groups that received a DOX formulation received an equivalent dose of 10 mg/kg DOX.

Twelve days after GL261-Luc cell implantation into the mouse brains, local intracerebral injection of the appropriate material was performed once every 8 days, for a total of three injections. The tumor sizes were observed via in vivo imaging. Moreover, the survival duration of each group of in situ GBM model mice was recorded. Fifty days after intracerebral injection of GL261-Luc cells, the animal experiment was terminated, and survival curves were constructed with Prism 8.0. After the experiment, the brains were carefully removed, and whole-brain paraffin sections were prepared. Then, the expression of Ki67 in these GBM tissue sections was analyzed via immunofluorescence, and TUNEL staining was used to label the apoptotic cells. Hematoxylin and eosin (HE) were applied to stain sections for microscopy evaluation of the morphological changes in the GBM tissue. Finally, Western blotting was used to detect apoptosis- and EMT-related protein expression in GBM tissue.

### 2.15. In Vivo Safety Evaluation of DOX/RGD-CD@Gel

After the experiment, the in situ GBM model mice were euthanized, and blood was collected from each mouse. The serum urea nitrogen (BUN), serum creatinine (CREA), alanine aminotransferase (ALT), aspartate aminotransferase (AST), and lactate dehydrogenase (LDH) levels were measured, and organs, such as the heart and the kidneys, were isolated and stained with HE for morphological evaluation.

### 2.16. Statistical Analysis

All data are expressed as the means ± SD and were analyzed by GraphPad Prism 8.0 software. The t-test was used for comparison between the two groups; *p* < 0.05 was considered to be statistically significant between the two groups.

## 3. Results and Discussion

### 3.1. Characterization of DOX/RGD-CD

The chemical structure of RGD-CD was verified by the ^1^H NMR and IR spectra (Figures S2 and S3). In the ^1^H NMR results, in addition to the characteristic peaks of CD, the characteristic peaks of RGD also appeared in the RGD-CD compounds, indicating that the RGD-CD compounds were successfully synthesized. In the IR results, when the CD interacted with RGD, the specific vibrational peak position of the CD changed significantly, which further confirmed the successful synthesis of RGD-CD compounds. The chemical structure of the inclusion complex (DOX/RGD-CD) was verified by the ^1^H NMR and IR spectra (Figures S4 and S5). From the ^1^H NMR analysis (Figure S4), when DOX was encapsulated in the CD cavity, the aromatic protons of DOX (δ 7.8–8.2 ppm) exhibited a 0.2–0.5 ppm upfield shift, indicative of potential hydrogen bonding between the hydroxyl groups of DOX and CD. Concurrently, the methoxy group of DOX (δ 3.8 ppm) demonstrated a 0.2 ppm upfield shift, suggesting synergistic π–π stacking interactions and hydrogen bonding between the anthraquinone ring of DOX and the hydrophobic CD cavity. Collectively, these spectral shifts provide conclusive evidence for the successful encapsulation of DOX within the CD cavity, confirming the formation of a stable inclusion complex. The IR (Figure S5) results show that in the wavenumber range of 800 to 1900 cm^−1^, the expansion and contraction of the C=O bond on the DOX anthracene ring and the skeleton vibration of the DOX molecule could be observed. At the same time, the range of 800 to 1630 cm^−1^ corresponds to the characteristic peak of the skeleton structure of CD, while the interval between 3200 and 3400 cm^−1^ shows the vibrational absorption peak of the O–H bond in the CD molecule. The infrared spectra of the mechanical mixture of DOX and CD roughly show the superposition of the monomer spectra of the two. In contrast, the infrared absorption spectra of the DOX/CD inclusion complexes showed different characteristics. It is evident from the spectral diagram that the vibrational peaks of the carbonyl group were displaced, while the band intensities of the mechanical mixtures in the range of 800 to 1900 cm^−1^, especially the characteristic stretching vibration peaks such as C=O, were significantly reduced in the clathballs. This change indicates that the anthracycline molecular backbone vibration of DOX in the inclusion complexes are weakened, thus confirming the formation of the inclusion complexes. For the DOX/RGD-CD inclusion complexes, the infrared spectral characteristics were similar to those of the DOX/CD inclusion complexes. Overall, the original absorption peak intensity and peak shape of DOX in the inclusion complexes were significantly changed compared with DOX and its mechanical mixture. The peak strength decreased, and the peak shape became less sharp. These changes mean that the oscillating and bending vibrations of the guest molecule (DOX) were limited due to the formation of the inclusion complexes, and it is likely that the anthracycline group in DOX was inserted into the cavity of CD.

### 3.2. Cytotoxicity of DOX/RGD-CD

**Figure 1:**
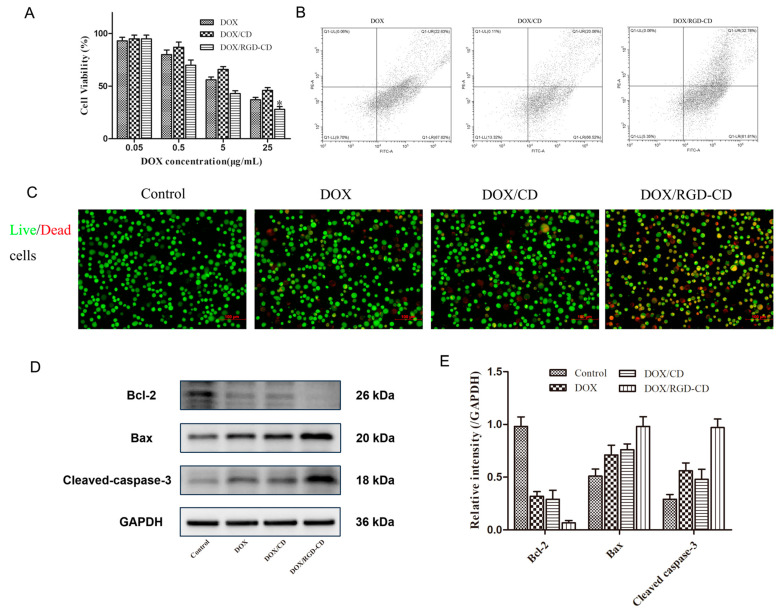
The effects of DOX/RGD-CD on GL261 cell activity and the underlying mechanisms. (**A**) The effect of DOX/RGD-CD on the cytotoxicity of GL261 cells. (**B**) The effect of DOX/RGD-CD on the apoptosis of GL261 cells. (**C**) The effect of DOX/RGD-CD on the live/death of GL261 cells. (**D**) The effect of DOX/RGD-CD on the expression of apoptosis-related proteins in GL261 cells. (**E**) The semi-quantitative statistical results of apoptosis-related protein expression (*n* = 3, $\bar{x}$ ± SD, * *p* < 0.05).

DOX/RGD-CD displayed dose-dependent toxicity to GL261 cells (Figure 1A). The IC_50_ values of free DOX, DOX/CD, and DOX/RGD-CD in GL261 cells were 11.16, 16.32, and 4.39 μg/mL, respectively. These results indicate that DOX/RGD-CD is more toxic to GL261 cells than DOX and DOX/CD. DOX, DOX/CD, and DOX/RGD-CD all effectively induced apoptosis of GL261 cells, and DOX/RGD-CD had a stronger effect on apoptosis than free DOX and DOX/CD (Figure 1B). Many dead cells were observed in each drug group in the live/dead cell staining assay. These experimental results were similar to those of the MTT assay, wherein the greatest number of dead cells was observed in the DOX/RGD-CD treatment group, indicating that it had the strongest cell-killing effect (Figure 1C). Western blotting revealed that DOX/RGD-CD significantly reduced the expression of Bcl-2 in GL261 cells and significantly increased the expression of cleaved caspase-3 and Bax (Figure 1D,E). These results indicate that DOX/RGD-CD inhibits the proliferation of GL261 cells by activating the caspase-dependent apoptosis pathway.

### 3.3. Effects of DOX/RGD-CD on GL261 Cell Migration and Invasion

**Figure 2:**
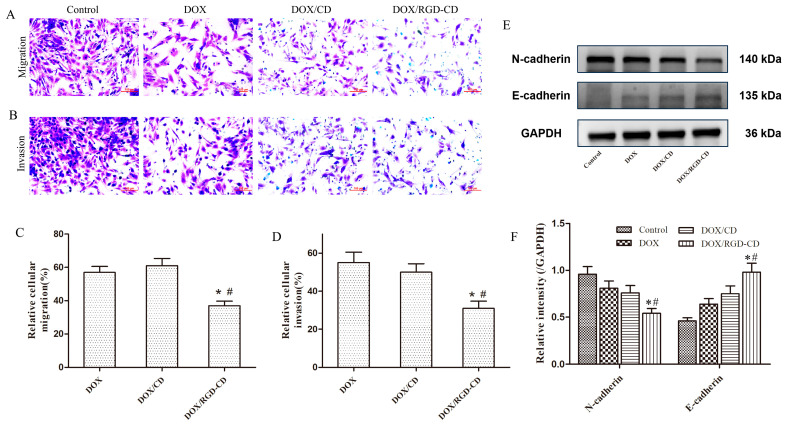
The effects of DOX/RGD-CD on the migration and invasion of GL261 cells. (**A**) The effect of DOX/RGD-CD on the migration of GL261 cells. (**B**) The effect of DOX/RGD-CD on the invasion of GL261 cells. (**C**) The relative migration rate of GL261 cells after the treatment with DOX/RGD-CD. (**D**) The relative invasion rate of GL261 cells after the treatment with DOX/RGD-CD. (**E**) The effect of DOX/RGD-CD on the expression of invasion-related proteins in GL261 cells. (**F**) Semi-quantitative statistical results of the invasion-related proteins. (*n* = 3, $\bar{x}$ ± SD, *^,#^ *p* < 0.05).

GL261 cells had strong migration and invasion capabilities, whereas DOX, DOX/CD, and DOX/RGD-CD significantly inhibited the migration and invasion of GL261 cells (Figure 2A–D). DOX/RGD-CD exhibited a higher inhibitory effect on the migration and invasion of GL261 cells compared to the same concentration of DOX and DOX/CD. It is well-known that migration and invasion of tumor cells are closely related to the occurrence of EMT [27,28]. Thus, EMT-related proteins were then detected by Western blot (Figure 2E,F). Compared with DOX and DOX/CD, DOX/RGD-CD treatment led to a significant increase in the expression of E-cadherin and a decrease in the expression of N-cadherin in GL261 cells. E-cadherin is a key molecule that maintains intercellular adhesion in cancer cells, and it is generally believed that the loss of or a reduction in E-cadherin expression in cancer cells is closely related to their invasion and metastatic capabilities [29,30]. In addition, N-cadherin can be upregulated in cancer cells, thereby enhancing their motility. These results indicated that DOX/RGD-CD reduced the migration and the invasion ability of GL261 cells by increasing the expression of E-cadherin and decreasing the expression of N-cadherin.

### 3.4. Uptake of DOX/RGD-CD by GL261 Cells

**Figure 3:**
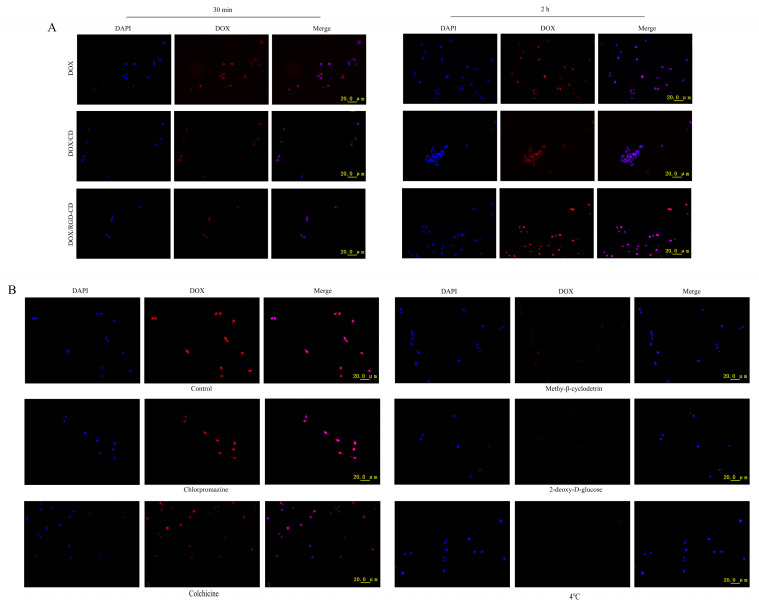
Cellular uptake and uptake mechanism of DOX/RGD-CD by GL261 cells. (**A**) The uptake of DOX/RGD-CD by GL261 cells observed via CLSM. (**B**) The effect of uptake inhibitors on the uptake of DOX/RGD-CD by GL261 cells observed via CLSM.

The accumulation of DOX/RGD-CD in GL261 cells was significantly greater than that of free DOX or DOX/CD. As the culture duration increased, the accumulation of DOX/RGD-CD in GL261 cells significantly increased (Figure 3A and Figure S6). After GL261 cells were cultured with free DOX, DOX/CD, or DOX/RGD-CD, the intracellular accumulation of DOX was also evaluated via HPLC. Compared with that in the DOX/CD group, the accumulation of DOX in the free DOX group was significantly greater after 15 min. However, after 1.5 h of culture, significantly more DOX had accumulated in the GL261 cells in the DOX/RGD-CD group than in the free DOX group (Figure S7). In addition, throughout the experiment, the accumulation of DOX in the DOX/RGD-CD group was significantly greater than that in the free DOX and DOX/CD groups. These findings confirmed that modification with RGD enhanced the uptake of DOX/RGD-CD by GL261 cells. In addition, DOX/RGD-CD uptake by GL261 cells was significantly reduced after methyl-β-cyclodextrin, colchicine, and 2-deoxy-D-glucose pretreatment and treatment at low temperatures (Figure 3B and Figure S8), indicating that GL261 cells take up DOX/RGD-CD via caveolin-mediated endocytosis and macropinocytosis [31,32].

### 3.5. Characterization of DOX/RGD-CD@Gel

**Figure 4:**
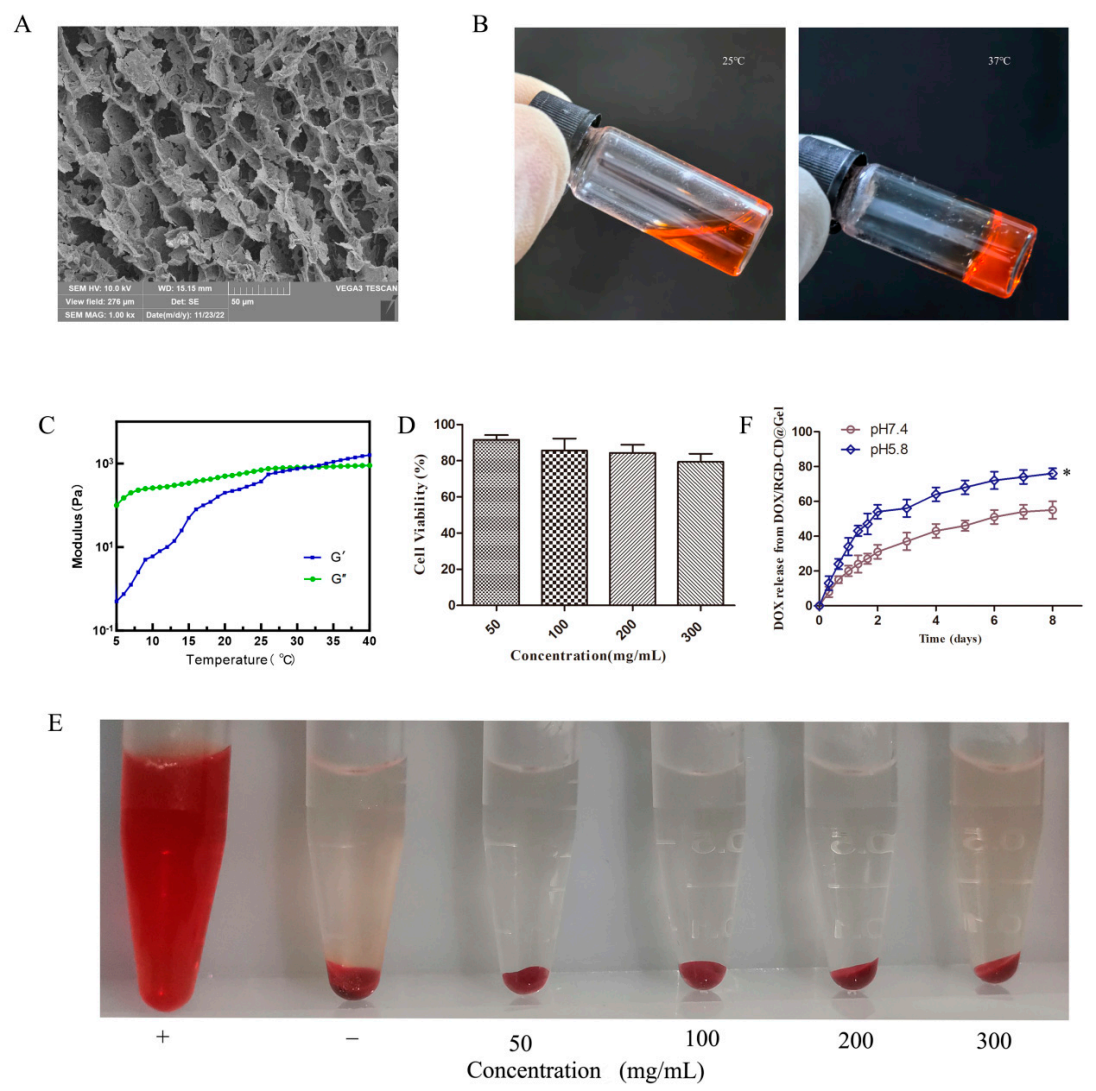
Physicochemical characterization of DOX/RGD-CD@Gel. (**A**) Representative SEM image of DOX/RGD-CD@Gel. (**B**) The temperature-responsive phase transition process of DOX/RGD-CD@Gel. (**C**) Rheological characterization of DOX/RGD-CD@Gel. (**D**) Cytotoxicity of Blank@Gel. (**E**) Erythrocyte hemolysis of Blank@Gel. (**F**) In vitro DOX release from DOX/RGD-CD@Gel in different pH release media (*n* = 3, $\bar{x}$ ± SD, * *p* < 0.05).

DOX/CD and DOX/RGD-CD were embedded into the thermosensitive hydrogel framework Blank@Gel to construct DOX/CD@Gel and DOX/RGD-CD@Gel, respectively. The micromorphology of the DOX/CD@Gel and DOX/RGD-CD@Gel hydrogels was observed using scanning electron microscopy. The results (Figure 4A and Figure S9) revealed that both the DOX/CD@Gel and DOX/RGD-CD@Gel hydrogels exhibited a three-dimensional network structure with a porous skeleton that was capable of loading water and drug molecules. The heat-induced sol-gel phase transition abilities of the hydrogels were then verified, as shown in Figure 4B and Figure S10. DOX/CD@Gel and DOX/RGD-CD@Gel were liquids at room temperature, whereas their solutions became semisolid after heating at 37 °C. The moduli of the DOX/CD@Gel and DOX/RGD-CD@Gel were further determined using a dynamic mechanical analyzer. As the temperature increased from 5 °C to 40 °C, the storage modulus (G′) of the solution increased from 3.64 to 972.5 Pa. Then, G′ passed the loss modulus (G″) at 33.8 °C, indicating that the elastic component exceeded the viscous component, which corresponds to the solution-to-gel transition (Figure 4C and Figure S11). These results indicate that DOX/CD@Gel and DOX/RGD-CD@Gel have excellent temperature sensitivity and can serve as excellent drug reservoirs for postoperative intracavitary injection for GBM treatment.

The in vitro biocompatibility of different concentrations (50, 100, 200, and 300 mg/mL) of Blank@Gel was determined with HUVEC cells. As shown in Figure 4D, there was no significant difference in the toxicity of the different concentrations of Blank@Gel to HUVEC cells. The hemolysis rate of Blank@Gel was less than 5% (4.1%), and the visual appearance of the sample solution was the same as that of the negative group but significantly different from that of the positive group (Figure 4E and Figure S12), indicating that this hydrogel system does not cause hemolysis and has potential for use as a qualified medical material. Therefore, the hydrogel can be safely loaded with drugs and ultimately injected into the cavity of a mouse brain tumor.

When evaluating the overall safety and effectiveness of a carrier system, the kinetics of drug release is a crucial factor that requires primary consideration [33,34]. To simulate the physiological environment of the human body and the acidic environment of tumour cells, DOX was placed in fresh phosphate buffer solutions at pH 5.8 and 7.4 and 37 °C for in vitro release experiments (Figure S13). After 48 hours at pH 5.8, the cumulative release of DOX was approximately 70%, whereas at pH 7.4, the cumulative release of DOX was approximately 53%. Thus, the cumulative release of DOX was relatively high at pH 5.8. One possible reason for this result is that in an acidic environment (pH 5.8), the amino groups on the DOX molecules may combine with hydrogen ions in the acidic buffer solution to form DOX salts, which are more soluble under acidic conditions [35]. Owing to the increased solubility of DOX under acidic conditions, DOX release also increased with decreasing environmental pH [36]. Therefore, these experimental results demonstrate that DOX release is pH responsive, making this system applicable for releasing DOX within the acidic environment of tumour cells. The long-term release experimental results with DOX/RGD-CD@Gel are shown in Figure 4F. At pH 5.8, more than 30% of the free DOX was released within 24 hours, and the cumulative amount released within 8 days was >75%. In contrast, only 45% of the DOX was released from DOX/RGD-CD@Gel within 8 days. The drug release properties of DOX/CD@Gel are similar to those of DOX/RGD-CD@Gel (Figure S14). These findings indicate that both the DOX/CD@Gel and DOX/RGD-CD@Gel system releases the DOX in a slow and sustained manner, making it a promising carrier for local treatment.

### 3.6. Antitumor Efficacy of DOX/RGD-CD@Gel In Vivo

**Figure 5:**
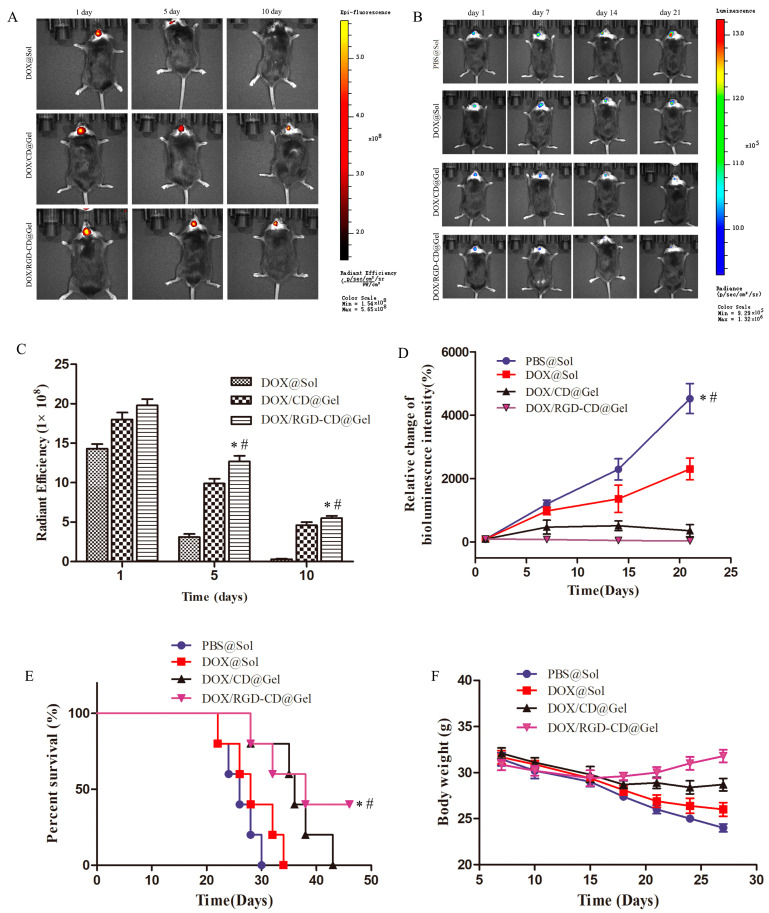
The biodistribution and therapeutic effect of DOX/RGD-CD@Gel in orthotopic GBM mice. (**A**) In vivo distribution of different formulations over time. (**B**) Semi-quantitative statistical results of fluorescence intensity in A. (**C**) The inhibitory effect of DOX/RGD-CD@Gel on the growth of orthotopic GBM observed by in vivo bioluminescence imaging. (**D**) Statistical analysis of orthotopic GBM growth. (**E**) Survival curve of orthotopic GBM mice. (**F**) Effects of DOX/RGD-CD@Gel on the body weight of orthotopic GBM mice (*n* = 6, $\bar{x}$ ± SD, *^,#^ *p* < 0.05).

In order to thoroughly investigate the retention of DOX@Sol, DOX/CD@Gel, and DOX/RGD-CD@Gel in the brains of mice, we injected them and tracked their movements using an in vivo imaging technology. As shown in Figure 5A,B, there were significant differences in the drug retention durations in the brain after the administration of the different drug delivery systems. Specifically, the fluorescence signal in the mouse brain was still clearly visible on day 10 in the DOX/CD@Gel and DOX/RGD-CD@Gel groups, indicating that the drug was stably released at the site of administration to be continuously effective. This result is important in terms of prolonging the effective treatment duration of the drug, reducing the frequency of administration, and improving patient compliance [37,38]. By contrast, the fluorescence signal observed in the DOX@Sol solution group nearly vanished by the fifth day, suggesting a swift diffusion and elimination of the drug from the brain. The aforementioned experimental results not only vividly illustrate the prolonged retention of DOX/CD@Gel and DOX/RGD-CD@Gel within the mouse brain, but also underscore the considerable promise of this system as an innovative drug delivery platform. As documented in the literature [39], hydrogels, due to their distinctive physicochemical properties, possess the ability to establish stable depots within the body. This capacity enables them to facilitate slow drug release, thereby markedly extending the duration of the drug’s activity. Notably, although the retention durations of DOX/RGD-CD@Gel and DOX/CD@Gel were similar, modification with RGD increases the potential for the specific recognition of and binding to tumor cells due to the targeting effects of this peptide.

To study the therapeutic effect of DOX/RGD-CD@Gel on glioblastoma in vivo, glioma-bearing mice were randomly divided into the PBS@Sol, DOX@Sol, DOX/CD@Gel, and DOX/RGD-CD@Gel groups. As shown in Figure 5C, each group was inoculated with GL261-Luc cells, which could be detected via bioluminescence, which represents the size of the tumor. Twelve days after inoculation, bioluminescence was detected in all groups, indicating successful tumor implantation. In addition, on day 12, the different formulations were administered to the mice. As shown in Figure 5C,D, compared with those of the other groups, the bioluminescence intensity on day 21 was the most intense in the PBS@Sol group, whereas that in the DOX/RGD-CD@Gel group was the weakest, indicating that DOX/RGD-CD@Gel had the best inhibitory effect on glioma cells. Survival curve analysis revealed the significantly prolonged survival duration of the mice in the DOX/RGD-CD@Gel treatment group, with a median survival time of 41 days. The median survival times of the other treatment groups were 23 days for the PBS@Sol group, 25 days for the DOX@Sol group, and 32 days for the DOX/CD@Gel group (Figure 5E). These results indicate that the DOX/RGD-CD@Gel hydrogel system can effectively reduce glioblastoma growth, thereby prolonging mouse survival. During treatment, the tumors in each group showed varying degrees of growth. Owing to the increasing tumor burden and the impact of tumor-related cachexia, the weights of the mice in each group generally gradually decreased. However, compared with those of the other treatment groups, the mice treated with DOX/RGD-CD@Gel displayed a more moderate weight loss (Figure 5F), suggesting that this drug delivery system may have certain advantages in terms of maintaining the overall health status of the mice.

**Figure 6:**
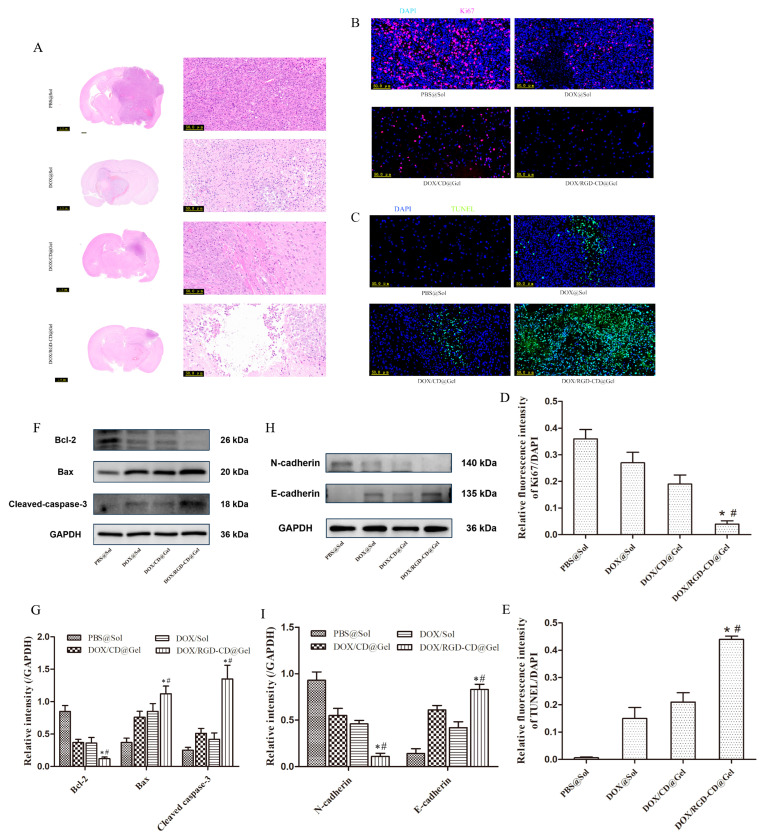
Effect of DOX/RGD-CD@Gel on the apoptosis and invasive microenvironment in orthotopic GBM tissue. (**A**) The HE staining of orthotopic GBM tissue slices. (**B**) Expression of Ki-67 staining of orthotopic GBM tissue. (**C**) Expression of TUNEL staining of orthotopic GBM tissue. (**D**) Semi-quantitative statistical results of Ki-67 expression. (**E**) Semi-quantitative statistical results of TUNEL staining. (**F**) The effect of DOX/RGD-CD@Gel on the expression of apoptosis-related proteins in orthotopic GBM tissue. (**G**) Semi-quantitative statistical results of apoptosis-related proteins. (**H**) The effect of DOX/RGD-CD@Gel on the expression of EMT-related protein in orthotopic GBM tissue. (**I**) Semi-quantitative statistical results of EMT-related proteins (*n* = 3, $\bar{x}$ ± SD, *^,#^ *p* < 0.05).

On the thirtieth day of the treatment period, we procured brain tumor tissues from each group to evaluate the proliferation and apoptosis of tumor cells. The HE staining of intact brain tissue from mice with orthotopically-implanted glioblastoma multiforme (GBM) is illustrated in Figure 6A. The tumor areas in the DOX@Sol, DOX/CD@Gel, and DOX/RGD-CD@Gel treatment groups were notably smaller than those in the PBS@Sol treatment group, with the smallest tumor area observed in the DOX/RGD-CD@Gel group. Additionally, the density of GBM cells in the DOX/RGD-CD@Gel treatment group was lower than that in the PBS@Sol, DOX@Sol, and DOX/CD@Gel treatment groups. In the PBS@Sol and DOX@Sol groups, the tumor regions exhibited abnormal nuclear morphologies and a high incidence of pathological mitoses. However, compared to the PBS@Sol and DOX@Sol groups, the number of atypical cells was significantly reduced in the DOX/CD@Gel and DOX/RGD-CD@Gel treatment groups. Furthermore, the number of pathological mitotic cells was notably smaller in the DOX/RGD-CD@Gel treatment group compared to the DOX/CD@Gel group. Immunohistochemical analysis revealed that the proportion of Ki-67-positive cells decreased most significantly in the tumor tissues of the mice treated with DOX/RGD-CD@Gel (Figure 6B,D). Ki-67 is a key indicator of tumor cell proliferation, and its low expression directly reflects the effective control of tumor growth by DOX/RGD-CD@Gel [40]. Moreover, TUNEL staining was used to assess tumor cell apoptosis [41]. Compared with those in the other treatment groups, the tumor tissue in the DOX/RGD-CD@Gel treatment group presented significantly more green fluorescence, indicating a significantly greater proportion of apoptotic cells (Figure 6C,E). In summary, the DOX/RGD-CD@Gel treatment strategy not only effectively controlled tumor growth, but also effectively promoted tumor cell apoptosis.

The novel drug delivery system DOX/RGD-CD@Gel significantly affected the expression of proteins related to apoptosis and epithelial-mesenchymal transition (EMT) in the treatment of GBM. As shown in Figure 6F,G, compared with free DOX and DOX/CD@Gel, DOX/RGD-CD@Gel significantly downregulated the antiapoptotic protein Bcl-2, a change that is consistent with many previous studies [42]. Moreover, this drug system also upregulated the proapoptotic proteins cleaved caspase-3 and Bax, a dual effect that clearly indicates that DOX/RGD-CD@Gel successfully activated the caspase-dependent classical apoptosis pathway, laying a solid foundation for effectively inhibiting the growth of malignant GBM tissue. Notably, DOX/RGD-CD@Gel also demonstrated an extraordinary ability to regulate the expression of EMT-related proteins (Figure 6H,I). It significantly increased the expression of the epithelial marker E-cadherin, a key molecule in maintaining the polarity and stability of epithelial cells, and an increase in its expression is often associated with a decrease in the invasiveness and metastatic abilities of tumor cells [43]. Conversely, DOX/RGD-CD@Gel effectively reduced the expression of the mesenchymal marker N-cadherin, wherein N-cadherin overexpression is usually considered a marker of tumor cells undergoing EMT and acquiring greater invasive potential [44]. Therefore, by upregulating E-cadherin and downregulating N-cadherin, DOX/RGD-CD@Gel effectively hindered the EMT process, thereby weakening the invasiveness and migratory abilities of GBM at the source, providing a new strategy for the treatment of GBM.

### 3.7. Preliminary Safety Evaluation of DOX/RGD-CD@Gel

**Figure 7:**
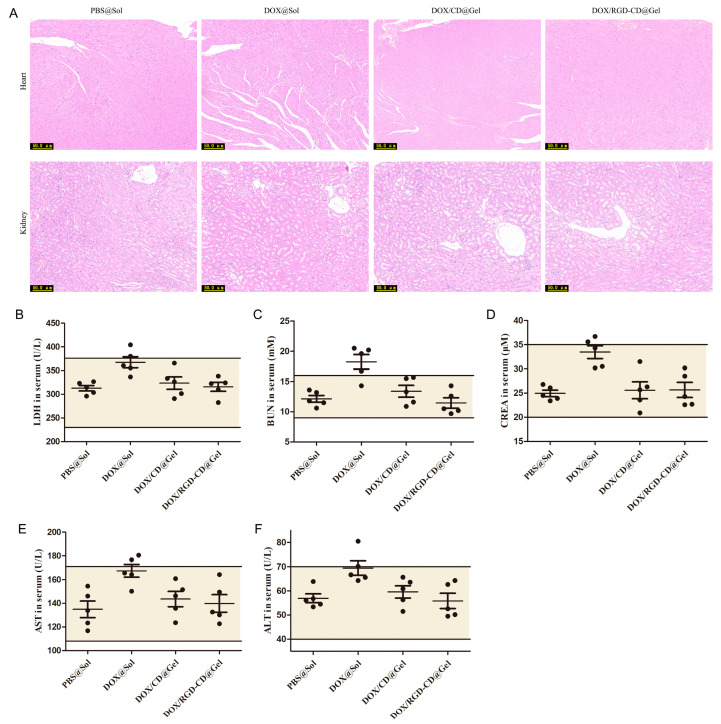
Preliminary safety evaluation of DOX/RGD-CD@Gel in mice. (**A**) HE staining of the heart and kidney tissue in mice. (**B**) Effects of DOX/RGD-CD@Gel on the content of LDH activity in serum of mice. (**C**,**D**) Effects of the DOX/RGD-CD@Gel on the contents of BUN and CREA in serum of mice. (**E**,**F**) Effects of DOX/RGD-CD@Gel on the content of ALT and AST activity in serum of mice. The earthy yellow area indicates the normal ranges (*n* = 3, $\bar{\bar{x}}$ ± SD).

HE staining clearly revealed that mice treated with free DOX had significant morphological abnormalities in their heart and kidneys, which is consistent with the reported side effects of DOX [45]. In contrast, no significant morphological abnormalities were observed in the hearts or kidneys of the mice treated with DOX/RGD-CD@Gel (Figure 7A), highlighting the significant advantage of this drug delivery system in terms of reducing DOX toxicity. Biochemical analysis (Figure 7B–F) further revealed that, compared with those in the free DOX-treated group, the activities of LDH, ALT, and AST in the serum of the mice in the DOX/CD@Gel- and DOX/RGD-CD@Gel-treated groups were within the normal physiological range. Moreover, the contents of BUN and CREA were also within normal ranges. These results verified that this drug delivery system protected the functions of the heart and the kidneys in mice. On the basis of these biochemical indicators, we confidently state that DOX/CD@Gel and DOX/RGD-CD@Gel do not cause significant systemic toxicity in mice at therapeutic doses.

## 4. Conclusions

In this study, a novel drug delivery system, DOX/RGD-CD@Gel, was developed for the treatment of glioblastoma multiforme (GBM). This system combines the advantages of the chemotherapeutic drug doxorubicin (DOX), the targeting ligand RGD, the carrier cyclodextrin (CD), and the thermosensitive hydrogel PLGA–PEG–PLGA. Experiments revealed that DOX/RGD-CD was significantly toxic to GBM cells, effectively inhibited GBM cell proliferation, migration, and invasion, and affected cell behavior by regulating the expression of apoptotic and EMT-related proteins. The DOX/RGD-CD@Gel system has good sensitivity to temperature and sustained drug release properties, releases the drug in acidic environments, and can be retained in the brain for a long time. In animal experiments, the system significantly inhibited tumor growth, prolonged mouse survival, and reduced the toxicity of DOX. Compared to other GBM drug delivery systems such as liposomes and nanoparticles, our DOX/RGD-CD@Gel system offers several advantages. Unlike liposomes and nanoparticles, which often suffer from premature drug release and rapid clearance from the body, our hydrogel system provides sustained drug release and prolonged drug retention at the tumor site. Additionally, the RGD modification in our system enhances specific targeting to GBM cells, potentially offering better therapeutic outcomes than non-targeted hydrogel systems. In conclusion, DOX/RGD-CD@Gel offers an innovative approach for the treatment of glioblastoma multiforme (GBM), exhibiting remarkable therapeutic efficacy and safety, thereby possessing significant potential for clinical application.

## Data Availability

The data supporting the findings of this study are available within the article and its ESI. Requests for access to additional data should be directed to the corresponding author.

## Supplementary Materials

The following supporting information can be downloaded at: https://www.mdpi.com/article/10.3390/pharmaceutics17070938/s1, Figure S1: Chemical structures of CM-β-CD, RGD, and DOX, Figure S2: ^1^H NMR spectrum of CD and RGD-CD, Figure S3: IR spectrum of CD and RGD-CD, Figure S4: IR spectrum of DOX, DOX/CD, DOX+CD, DOX/RGD-CD and DOX+RGD-CD, Figure S5: ^1^H NMR spectrum of DOX, DOX/CD and DOX/RGD-CD, Figure S6: Semi-quantitative analysis results of DOX, DOX/CD and DOX/RGD-CD uptake by GL261 cells, Figure S7: Cellular uptake of DOX, DOX/CD and DOX/RGD-CD by GL261 cells detected by HPLC, Figure S8: Semi-quantitative analysis results of DOX/RGD-CD uptake by GL261 cells, Figure S9: Representative SEM image of DOX/CD@Gel, Figure S10: The temperatureresponsive phase transition process of DOX/CD@Gel, Figure S11: Rheological characterization of DOX/CD@Gel, Figure S12: The hemolysis rate of Blank@Gel, Figure S13: In vitro DOX release from DOX@Sol in different pH release medium, Figure S14: In vitro DOX release from DOX/CD@Gel in different pH release medium.

## References

[1] Fine H.A. (2024). Glioblastoma: Not Just Another Cancer. Cancer Discov..

[2] Stepanenko A.A., Andreieva S., Korets K., Mykytenko D., Huleyuk N., Vassetzky Y., Kavsan V. (2024). Systemic and local immunosuppression in glioblastoma and its prognostic significance. Front. Immunol..

[3] Stahl P., Henoch I., Smits A., Rydenhag B., Ozanne A. (2022). Quality of life in patients with glioblastoma and their relatives. Acta Neurol. Scand..

[4] Obrador E., Moreno-Murciano P., Oriol-Caballo M., Lopez-Blanch R., Pineda B., Gutierrez-Arroyo J.L., Loras A., Gonzalez-Bonet L.G., Martinez-Cadenas C., Estrela J.M. (2024). Glioblastoma Therapy: Past, Present and Future. Int. J. Mol. Sci..

[5] Roda D., Veiga P., Melo J.B., Carreira I.M., Ribeiro I.P. (2024). Principles in the Management of Glioblastoma. Genes.

[6] Hu M., Liu H., Zhang Y., Lu D., Zheng L., Wang Y., Chen S., Liu T. (2024). Preparation and evaluation of the PD0721-DOX antibody-drug conjugate targeting EGFRvIII to inhibit glioblastoma. Exp. Ther. Med..

[7] Shimia M., Amini M., Ravari A.O., Tabnak P., Valizadeh A., Ghaheri M., Yousefi B. (2024). Thymoquinone reversed doxorubicin resistance in U87 glioblastoma cells via targeting PI3K/Akt/mTOR signaling. Chem. Biol. Drug Des..

[8] Ali B.H., Khoee S., Mafakheri F., Sadri E., Mahabadi V.P., Karimi M.R., Shirvalilou S., Khoei S. (2024). Active targeted delivery of theranostic thermo/pH dual-responsive magnetic Janus nanoparticles functionalized with folic acid/fluorescein ligands for enhanced DOX combination therapy of rat glioblastoma. J. Mater. Chem. B.

[9] Dash B.S., Lu Y.J., Huang Y.S., Chen J.P. (2024). Chitosan-coated magnetic graphene oxide for targeted delivery of doxorubicin as a nanomedicine approach to treat glioblastoma. Int. J. Biol. Macromol..

[10] Koula G., Yakati V., Rachamalla H.K., Bhamidipati K., Kathirvel M., Banerjee R., Puvvada N. (2024). Integrin receptor-targeted, doxorubicin-loaded cerium oxide nanoparticles delivery to combat glioblastoma. Nanomedicine.

[11] Tian B., Liu Y., Liu J. (2020). Cyclodextrin as a magic switch in covalent and non-covalent anticancer drug release systems. Carbohydr. Polym..

[12] Feng L., Wei R., Wu J., Chen X., Wen Y., Chen J. (2024). Cyclodextrin Drugs in Liposomes: Preparation and Application of Anticancer Drug Carriers. AAPS PharmSciTech.

[13] Yuan Y., Nie T., Fang Y., You X., Huang H., Wu J. (2022). Stimuli-responsive cyclodextrin-based supramolecular assemblies as drug carriers. J. Mater. Chem. B.

[14] Viale M., Tosto R., Giglio V., Pappalardo G., Oliveri V., Maric I., Mariggio M.A., Vecchio G. (2019). Cyclodextrin polymers decorated with RGD peptide as delivery systems for targeted anti-cancer chemotherapy. Investig. New Drugs.

[15] Zhang S., Tamura A., Yui N. (2024). Enhanced Tumor Targeting and Antitumor Activity of Methylated beta-Cyclodextrin-Threaded Polyrotaxanes by Conjugating Cyclic RGD Peptides. Biomolecules.

[16] Kebebe D., Wu Y., Zhang B., Yang J., Liu Y., Li X., Ma Z., Lu P., Liu Z., Li J. (2019). Dimeric c(RGD) peptide conjugated nanostructured lipid carriers for efficient delivery of Gambogic acid to breast cancer. Int. J. Nanomed..

[17] Xu L., Zhou C., Wang F., Liu H., Dong G., Zhang S., Liu T. (2022). Functional drug carriers formed by RGD-modified beta-CD-HPG for the delivery of docetaxel for targeted inhibition of nasopharyngeal carcinoma cells. RSC Adv..

[18] Kottmann V., Kolpeja E., Baumkotter G., Clauder F., Bokel A., Armbruster F.P., Drees P., Gercek E., Ritz U. (2024). Bone sialoprotein stimulates cancer cell adhesion through the RGD motif and the alphavbeta3 and alphavbeta5 integrin receptors. Oncol. Lett..

[19] Misra S.K., Kondaiah P., Bhattacharya S., Boturyn D., Dumy P. (2014). Co-liposomes comprising a lipidated multivalent RGD-peptide and a cationic gemini cholesterol induce selective gene transfection in alphavbeta3 and alphavbeta5 integrin receptor-rich cancer cells. J. Mater. Chem. B.

[20] Tang Q., Lu B., He J., Chen X., Fu Q., Han H., Luo C., Yin H., Qin Z., Lyu D. (2022). Exosomes-loaded thermosensitive hydrogels for corneal epithelium and stroma regeneration. Biomaterials.

[21] Luo J., Zhao X., Guo B., Han Y. (2023). Preparation, thermal response mechanisms and biomedical applications of thermosensitive hydrogels for drug delivery. Expert Opin. Drug Deliv..

[22] Chen H., Xu J., Sun J., Jiang Y., Zheng W., Hu W., Qian H. (2024). Recent advances on thermosensitive hydrogels-mediated precision therapy. Asian J. Pharm. Sci..

[23] Norouzi M., Firouzi J., Sodeifi N., Ebrahimi M., Miller D.W. (2021). Salinomycin-loaded injectable thermosensitive hydrogels for glioblastoma therapy. Int. J. Pharm..

[24] Anggelia M.R., Cheng H.Y., Lin C.H. (2024). Thermosensitive Hydrogels as Targeted and Controlled Drug Delivery Systems: Potential Applications in Transplantation. Macromol. Biosci..

[25] Wang Q., Zhang K., Weng W., Chen L., Wei C., Bao R., Adu-Frimpong M., Cao X., Yu Q., Shi F. (2022). Liquiritin-Hydroxypropyl-Beta-Cyclodextrin Inclusion Complex: Preparation, Characterization, Bioavailability and Antitumor Activity Evaluation. J. Pharm. Sci..

[26] Liu D., Cheng Y., Qiao S., Liu M., Ji Q., Zhang B.L., Mei Q.B., Zhou S. (2022). Nano-Codelivery of Temozolomide and siPD-L1 to Reprogram the Drug-Resistant and Immunosuppressive Microenvironment in Orthotopic Glioblastoma. ACS Nano.

[27] Lu T., Zheng C., Fan Z. (2022). Cardamonin suppressed the migration, invasion, epithelial mesenchymal transition (EMT) and lung metastasis of colorectal cancer cells by down-regulating ADRB2 expression. Pharm. Biol..

[28] Wang C., Liu B., Dan W., Wei Y., Li M., Guo C., Zhang Y., Xie H. (2024). Liquiritigenin inhibits the migration, invasion, and EMT of prostate cancer through activating ER stress. Arch. Biochem. Biophys..

[29] Sommariva M., Gagliano N. (2020). E-Cadherin in Pancreatic Ductal Adenocarcinoma: A Multifaceted Actor during EMT. Cells.

[30] Lu H., Cao L.L., Ballout F., Belkhiri A., Peng D., Chen L., Chen Z., Soutto M., Wang T.C., Que J. (2023). Reflux conditions induce E-cadherin cleavage and EMT via APE1 redox function in oesophageal adenocarcinoma. Gut.

[31] Pina A., Kadri M., Arosio D., Dal Corso A., Coll J.L., Gennari C., Boturyn D. (2020). Multimeric Presentation of RGD Peptidomimetics Enhances Integrin Binding and Tumor Cell Uptake. Chemistry.

[32] Yao P., Wang X., Wang Q., Dai Q., Peng Y., Yuan Q., Mou N., Lv S., Weng B., Wang Y. (2023). Cyclic RGD-Functionalized pH/ROS Dual-Responsive Nanoparticle for Targeted Breast Cancer Therapy. Pharmaceutics.

[33] Xu Y., Chen Z., Hao W., Yang Z., Farag M., Vong C.T., Wang Y., Wang S. (2024). Berberine and magnolol exert cooperative effects on ulcerative colitis in mice by self-assembling into carrier-free nanostructures. J. Nanobiotechnol..

[34] Gao S., Zheng H., Xu S., Kong J., Gao F., Wang Z., Li Y., Dai Z., Jiang X., Ding X. (2023). Novel Natural Carrier-Free Self-Assembled Nanoparticles for Treatment of Ulcerative Colitis by Balancing Immune Microenvironment and Intestinal Barrier. Adv. Healthc. Mater..

[35] Cao H., Yi M., Wei H., Zhang S. (2022). Construction of Folate-Conjugated and pH-Responsive Cell Membrane Mimetic Mixed Micelles for Desirable DOX Release and Enhanced Tumor-Cellular Target. Langmuir.

[36] Cao H., Lu Q., Wei H., Zhang S. (2022). Phosphorylcholine zwitterionic shell-detachable mixed micelles for enhanced cancerous cellular uptakes and increased DOX release. J. Mater. Chem. B.

[37] Amirthalingam S., Rajendran A.K., Moon Y.G., Hang N.S. (2023). Stimuli-responsive dynamic hydrogels: Design, properties and tissue engineering applications. Mater. Horiz..

[38] Liu X., Yang Y., Inda M.E., Lin S., Wu J., Kim Y., Chen X., Ma D., Lu T.K., Zhao X. (2021). Magnetic Living Hydrogels for Intestinal Localization, Retention, and Diagnosis. Adv. Funct. Mater..

[39] Hu Y., Yu B., Jia Y., Lei M., Li Z., Liu H., Huang H., Xu F., Li J., Wei Z. (2023). Hyaluronate- and gelatin-based hydrogels encapsulating doxycycline as a wound dressing for burn injury therapy. Acta Biomater..

[40] Banstola A., Poudel K., Emami F., Ku S.K., Jeong J.H., Kim J.O., Yook S. (2021). Localized therapy using anti-PD-L1 anchored and NIR-responsive hollow gold nanoshell (HGNS) loaded with doxorubicin (DOX) for the treatment of locally advanced melanoma. Nanomedicine.

[41] Biezunska-Kusiak K., Kulbacka J., Choromanska A., Rembialkowska N., Michel O., Saczko J. (2023). Evaluation of the Anticancer Activity of Calcium Ions Introduced into Human Breast Adenocarcinoma Cells MCF-7/WT and MCF-7/DOX by Electroporation. Pharmaceuticals.

[42] Sabokrouh A., Atabi F., Jassem R.M., Mohammadi R. (2020). The Anticancer Efficacy of Platinum Azidothymidin on Hepatocellular Carcinoma Via Affecting the Telomerase and the BcL-2 Genes Expression. J. Gastrointest. Cancer.

[43] Wang W., Dong L., Zhao B., Lu J., Zhao Y. (2018). E-cadherin is downregulated by microenvironmental changes in pancreatic cancer and induces EMT. Oncol. Rep..

[44] Noor S.I., Hoffmann M., Rinis N., Bartels M.F., Winterhalter P.R., Hoelscher C., Hennig R., Himmelreich N., Thiel C., Ruppert T. (2021). Glycosyltransferase POMGNT1 deficiency strengthens N-cadherin-mediated cell-cell adhesion. J. Biol. Chem..

[45] Li N., Zhang T., Wang R., Sun Y., Chu L., Lu X., Sun K. (2023). Homotypic targeted nanoplatform enable efficient chemoimmunotherapy and reduced DOX cardiotoxicity in chemoresistant cancer via TGF-beta1 blockade. J. Control. Release.

