# Supplementary material for: In Situ Targeting RGD-Modified Cyclodextrin Inclusion Complex/Hydrogel Hybrid System for Enhanced Glioblastoma Therapy
Source: Pharmaceutics. 2025 Jul 20;17(7):938. doi: 10.3390/pharmaceutics17070938 (PMC12298666; doi:10.3390/pharmaceutics17070938)
Supplement: Supplementary file 1 [file pharmaceutics-17-00938-s001.zip › pharmaceutics-3704815-supplementary.pdf]

Supplementary Material

Xiaofeng Yuan [1,2,3,†], Zhenhua Wang [3,†], Pengcheng Qiu [2], Zhenhua Tong [4], Bingwen Wang [2], Yingjian Sun [1], Xue Sun [4], Lu Sui [4], Haiqiang Jia [1], Jiajun Wang [1], Haifeng Tang [2,*] and Weiliang Ye [1,*]

[1] Department of Pharmaceutics, School of Pharmacy, Fourth Military Medical University, Xi'an 710032, China; xiaofeng012345@163.com (X.Y.); sunyingjian2024@163.com (Y.S.); 13233030861@163.com (H.J.); 15319782276@163.com (J.W.)

[2] Department of Chinese Materia Medical and Natural Medicines, School of Pharmacy, Fourth Military Medical University, Xi'an 710032, China; qpc023@fmmu.edu.cn (P.Q.); wbw134610@163.com (B.W.)

[3] Chinese People's Liberation Army Logistics Support Force No. 967 Hospital, Dalian 116021, China; huangxin0607@163.com

[4] General Hospital of Northern Theater Command, No. 83, Wenhua Road, Shenyang 110016, China; zhenhua_tong@163.com (Z.T.); sunxue88@163.com (X.S.); hxfhlyc666@163.com (L.S.)

* Correspondence: tanghf71@fmmu.edu.cn (H.T.); yaojixue@fmmu.edu.cn (W.Y.)

† These authors contributed equally to this work

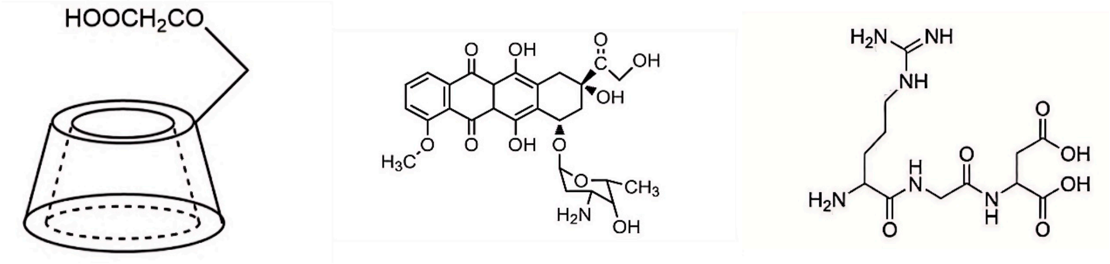

CM-β-CD DOX RGD

Figure S1. Chemical structures of CM-β-CD, RGD and DOX.

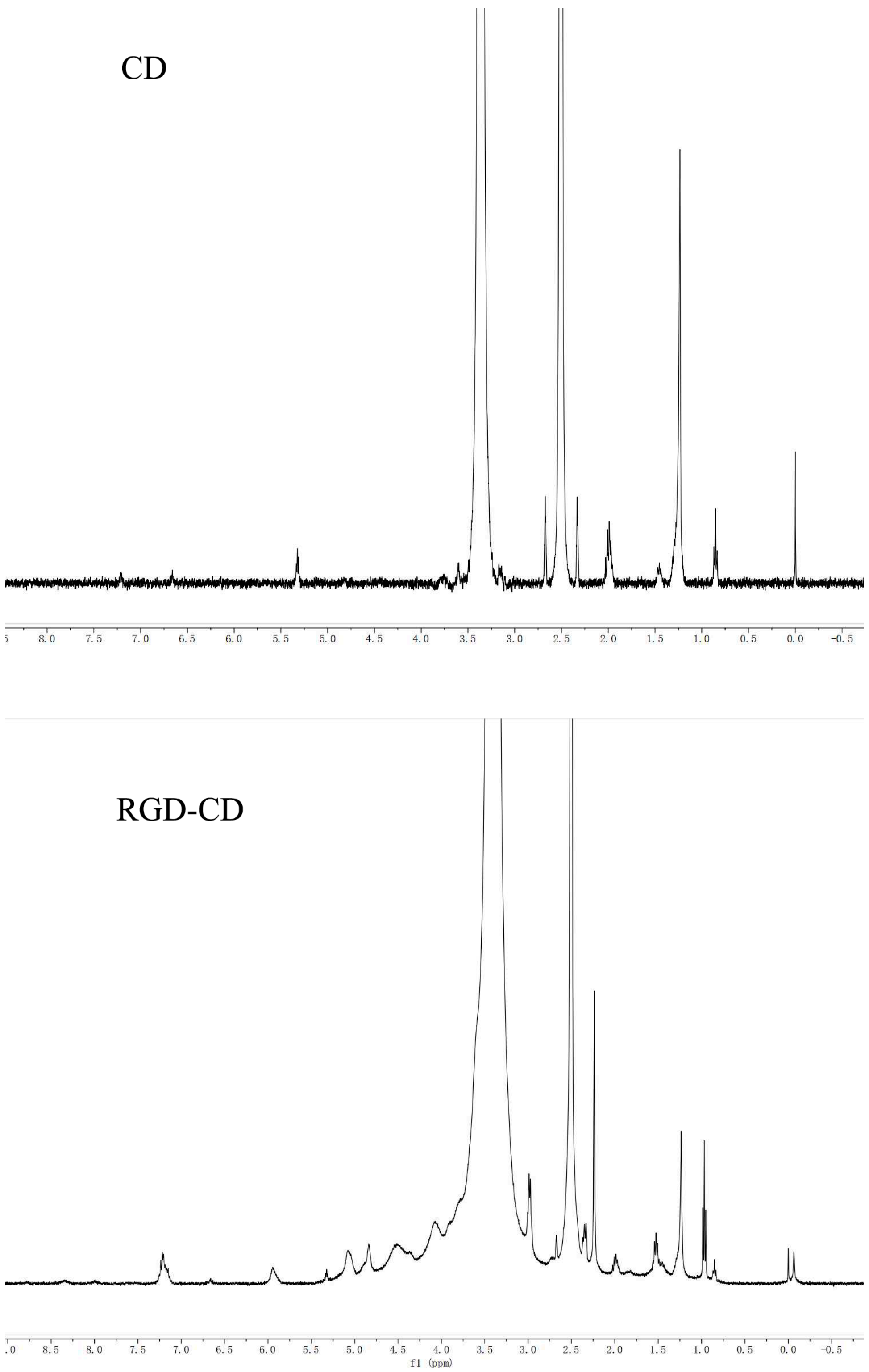

Figure S2. ^{1}H NMR spectrum of CD and RGD-CD.

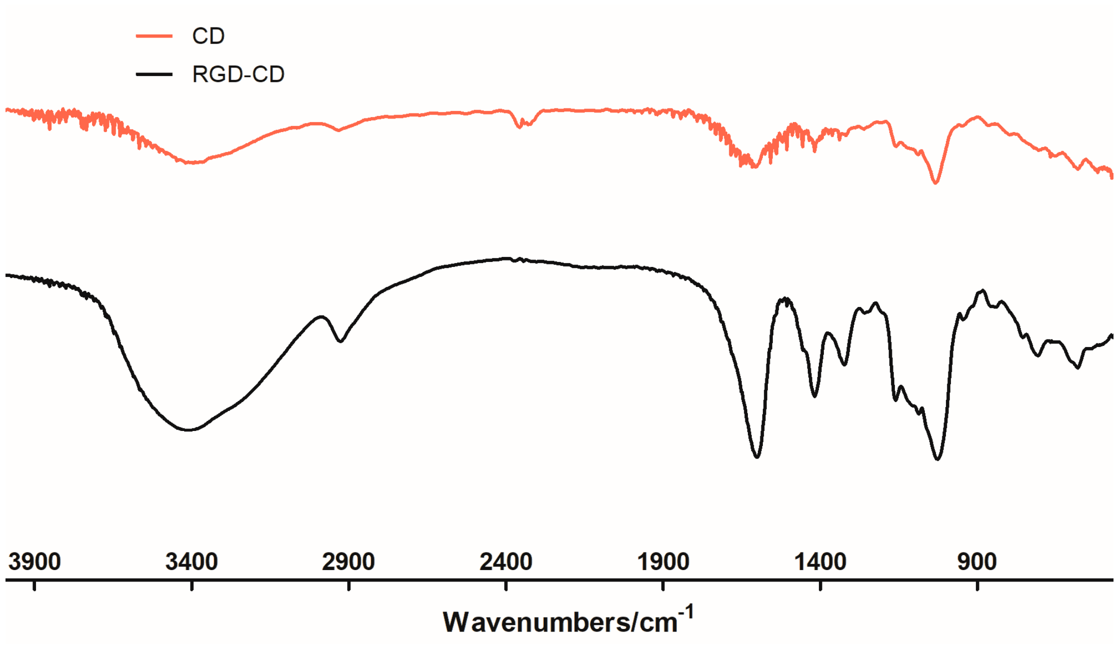

Figure S3. IR spectrum of CD and RGD-CD.

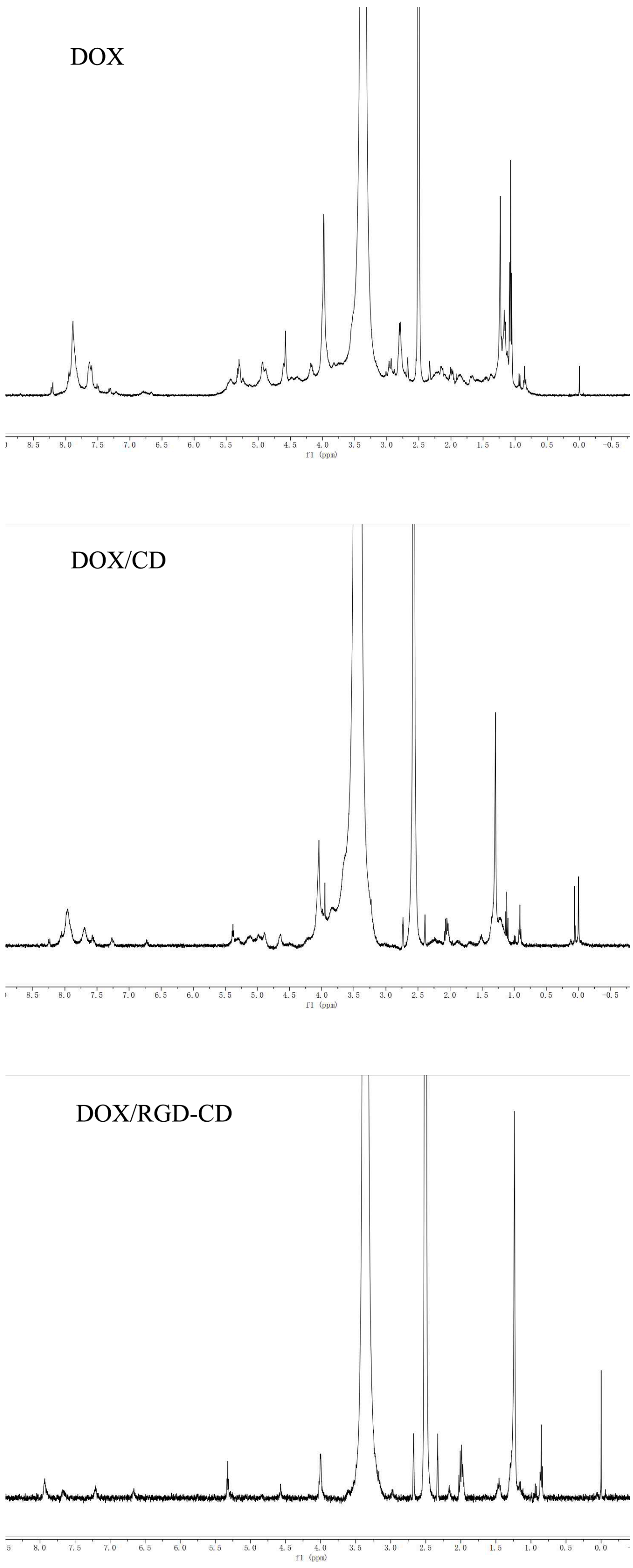

Figure S4. ^{1}H NMR spectrum of DOX、DOX/CD and DOX/RGD-CD.

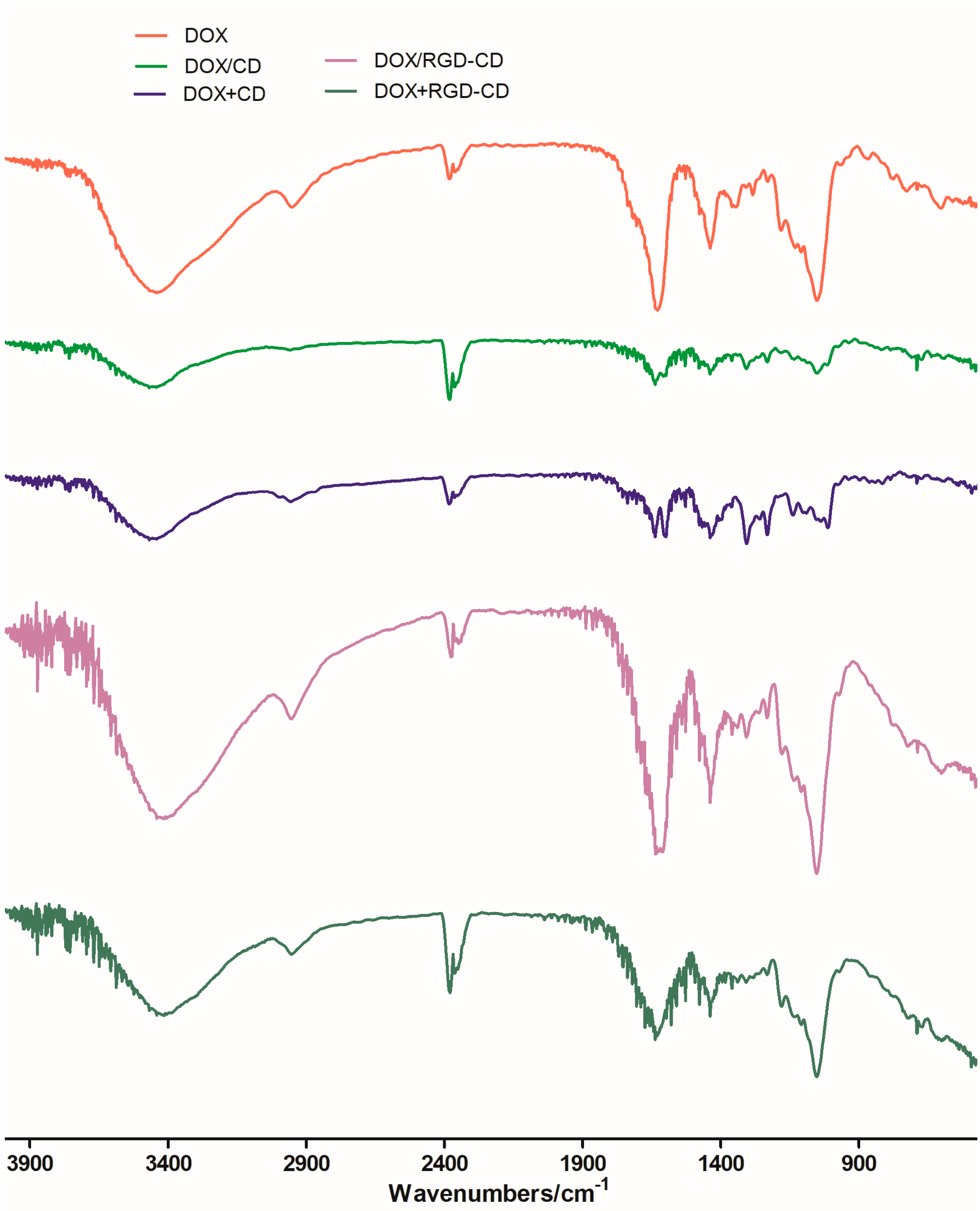

Figure S5. IR spectrum of DOX、DOX/CD、DOX+CD、DOX/RGD-CD and DOX+RGD-CD.

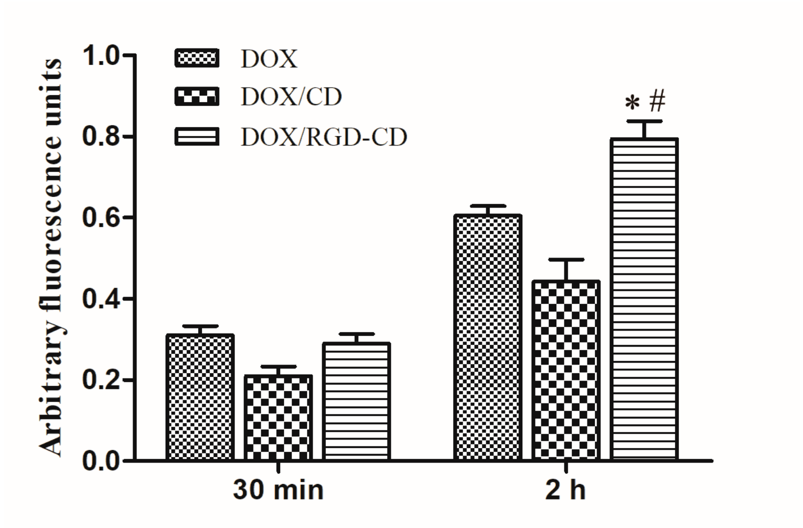

Figure S6. Semi-quantitative analysis results of DOX、DOX/CD and DOX/RGD-CD uptake by GL261 cells. (n=3, $\bar{x}$±SD, $^{*\#}P < 0.05$).

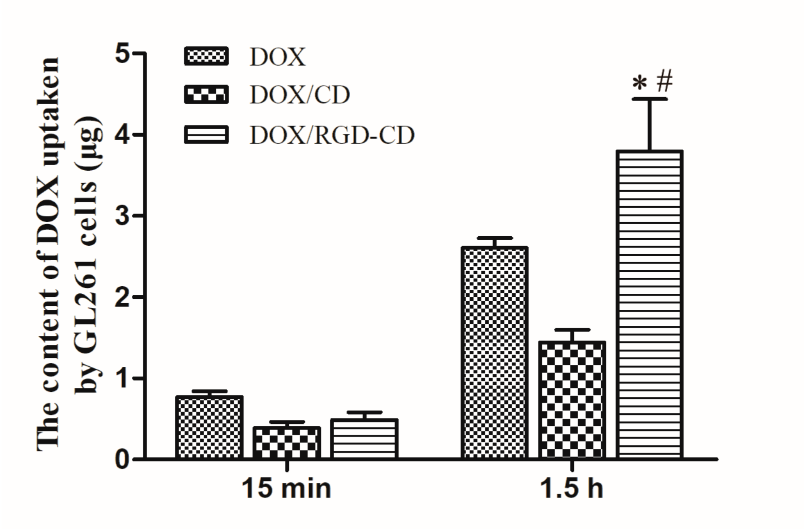

Figure S7. Cellular uptake of DOX、DOX/CD and DOX/RGD-CD by GL261 cells detected by HPLC (n=3, $\bar{x}$±SD, $^{*\#}P < 0.05$).

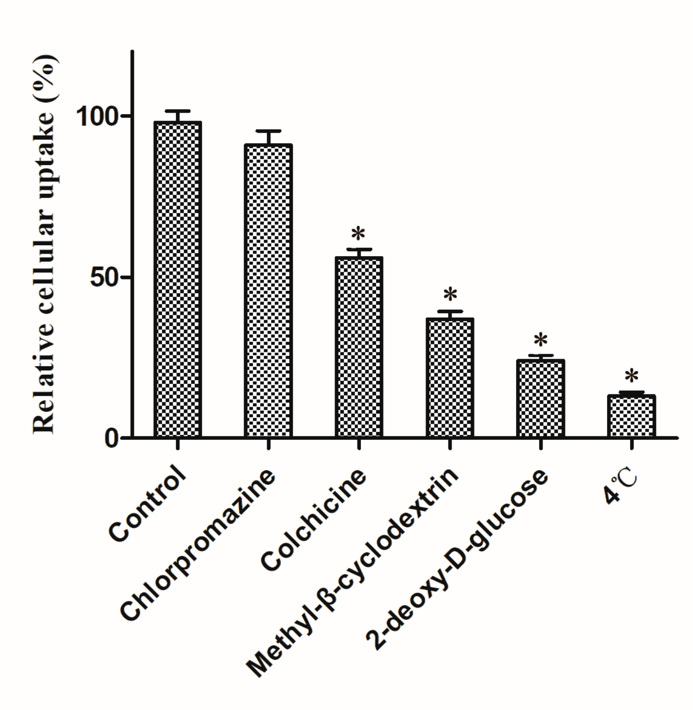

Figure S8. Semi-quantitative analysis results of DOX/RGD-CD uptake by GL261 cells. (n=3, x̄±SD, $^{*}P < 0.05$).

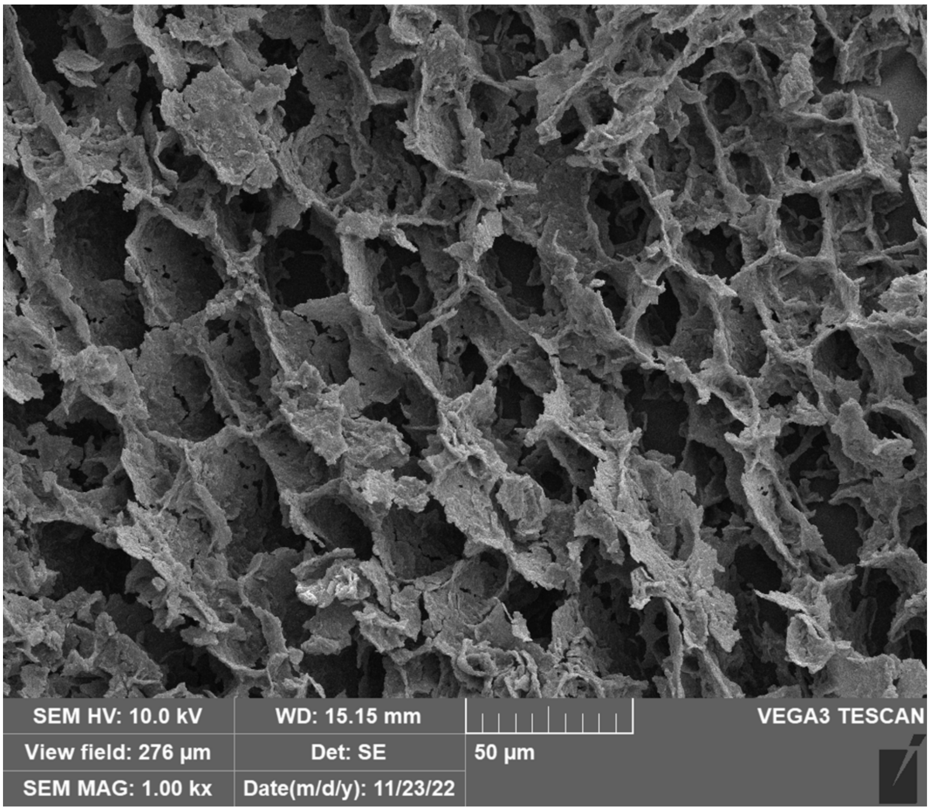

Figure S9. Representative SEM image of DOX/CD@Gel.

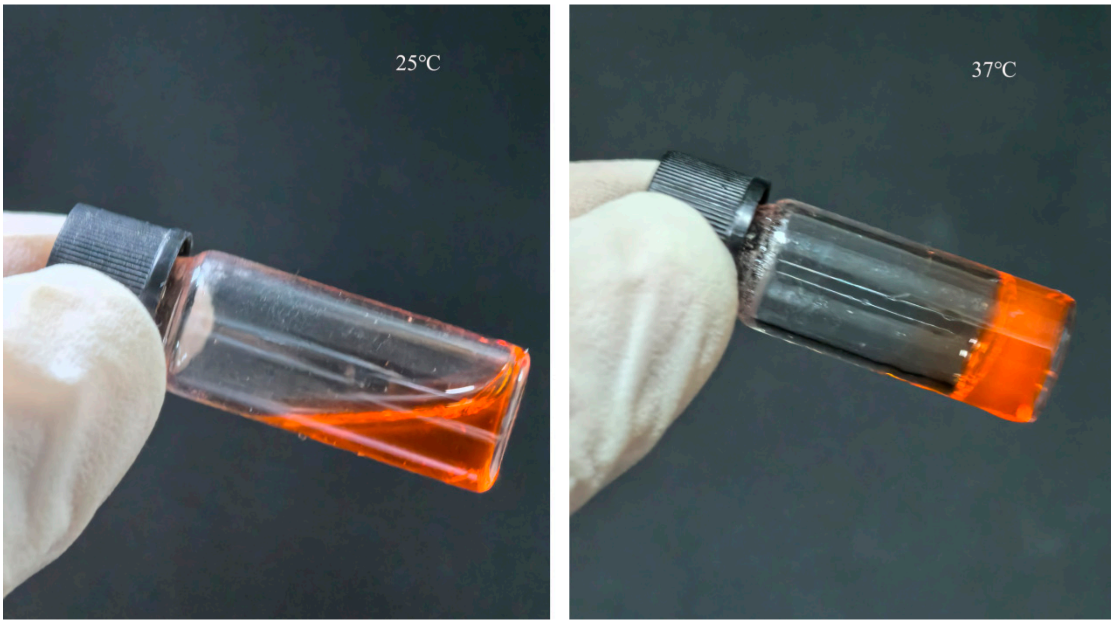

Figure S10. The temperatureresponsive phase transition process of DOX/CD@Gel.

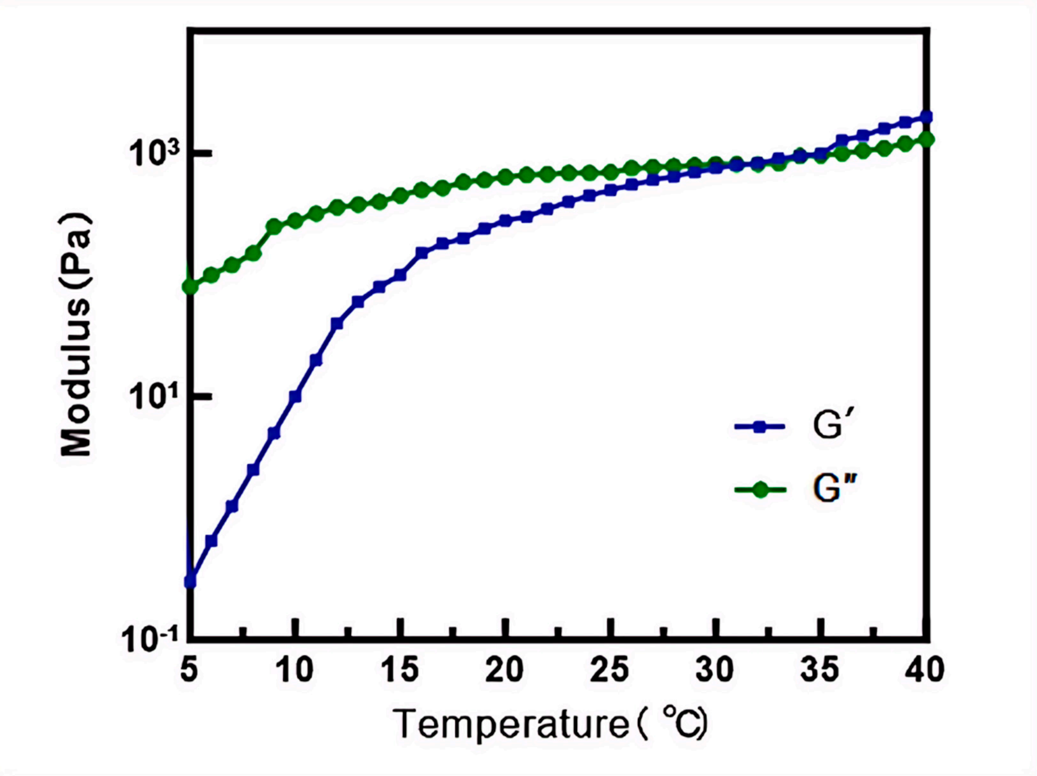

Figure S11. Rheological characterization of DOX/CD@Gel.

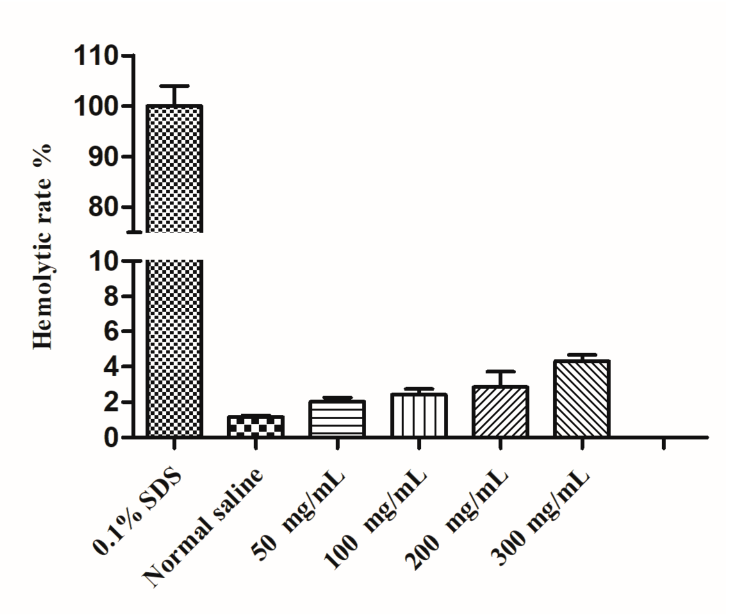

Figure S12. The hemolysis rate of Blank@Gel. (n=3, $\bar{x}$±SD).

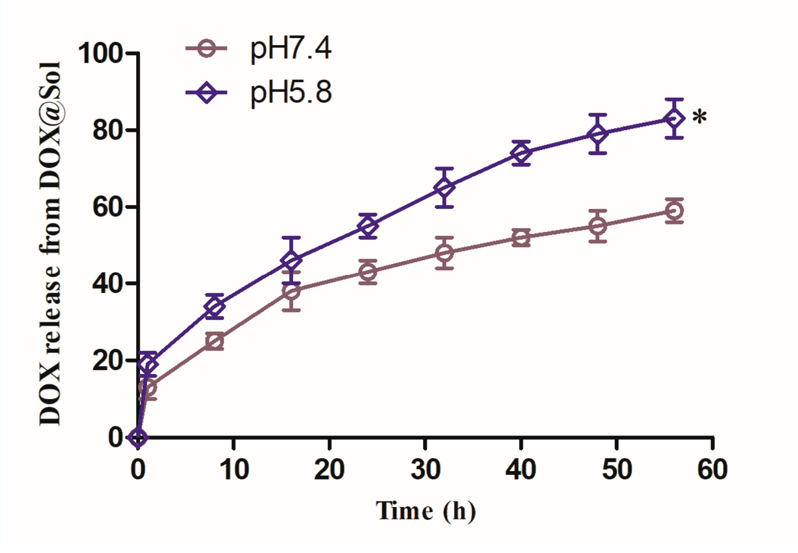

Figure S13. In vitro DOX release from DOX@Sol in different pH release medium. (n=3, $\bar{x}$±SD, $^{*}P < 0.05$).

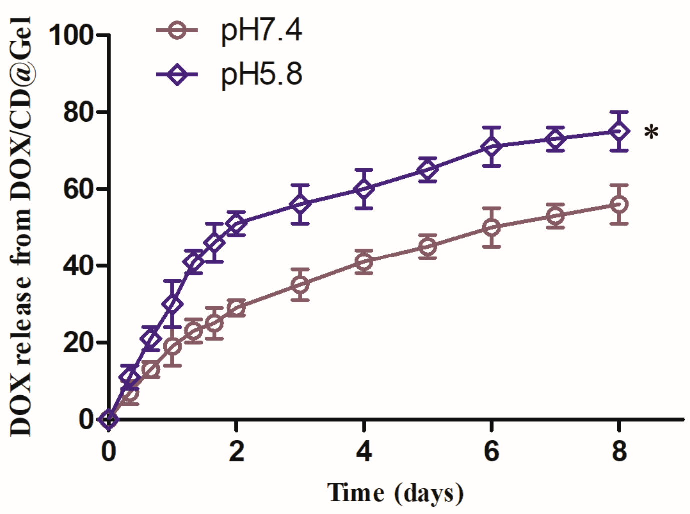

Figure S14. In vitro DOX release from DOX/CD@Gel in different pH release medium. (n=3, $\bar{x}$±SD, $^{*}P<0.05$).
